# Fibroblast growth factor 2 is necessary for the antidepressant effects of fluoxetine

**DOI:** 10.1371/journal.pone.0204980

**Published:** 2018-10-01

**Authors:** Stephanie Simard, Pragya Shail, Jessica MacGregor, Maha El Sayed, Ronald S. Duman, Flora. M. Vaccarino, Natalina Salmaso

**Affiliations:** 1 Department of Neuroscience, Carleton University, Ottawa, Ontario, Canada; 2 Department of Psychiatry, Yale University, New Haven, Connecticut, United States of America; 3 Child Study Center, Yale University, New Haven, Connecticut, United States of America; Radboud University Medical Centre, NETHERLANDS

## Abstract

Previous research has shown that fibroblast growth factor 2 protein (FGF2) can act as an anxiolytic and anti-depressive agent in rodents. Levels of hippocampal FGF2 and FGF2 receptors are decreased in post-mortem brains of individuals with mood disorders. No changes in FGF2 were noted in the post-mortem brains of individuals with mood disorders that were successfully treated with anti-depressant medication prior to death. Mutations in the *FGF2* gene in humans have been shown to predict non-responsiveness to the therapeutic effects of selective serotonin reuptake inhibitors (SSRIs). These findings suggest that FGF2 may potentially be a target of and/or required for the therapeutic effects of antidepressant medications. To test this, we employed a rodent model of depressive behaviour, chronic variable stress (CVS) in conjunction with antidepressant treatment (fluoxetine) in wild-type (WT) and *FGF2* knockout mice (FGF2KO) and examined depressive and anxiety behaviors. Results showed that fluoxetine reversed the effects of CVS on depressive and anxiety behaviours in wild-type mice only, suggesting that the FGF2 gene is indeed necessary for the therapeutic effects of fluoxetine. Interestingly, CVS decreased hippocampal FGF2 levels and fluoxetine partially reversed this effect. Because FGF2 has been previously shown to modify HPA activity through hippocampal glucocorticoid receptors (GR), we examined levels of glucocorticoid receptors and found a decrease in GR in response to CVS, with a further decrease in FGF2KO. No effect of fluoxetine on GR was observed in either WT or FGF2KO mice. This suggests that further changes in glucocorticoid receptors are not necessary for the anti-depressant effects of fluoxetine in WT mice, although decreased glucocorticoid receptors in response to *FGF2* deletion may preclude the therapeutic actions of fluoxetine in FGF2KO. Whether astroglia, astroglial functions, or HPA changes are the downstream target of FGF2-mediated changes induced by fluoxetine remains to be determined, however, the current study reaffirms the potential of FGF2 as a novel therapeutic target in the treatment of depression and anxiety disorders.

## Introduction

Mood and anxiety disorders currently are the most prevalent mental illnesses diagnosed in North America and have far-reaching effects on family and community function, as well as gastrointestinal, respiratory and cardiovascular diseases [1, 2]. While distinct, depressive and anxiety disorders have several overlapping symptoms and are often co-morbid. As such, current treatments are often similar for both disorders and range from cognitive-behavioral psychotherapy to antidepressant drugs such as selective serotonin reuptake inhibitors (SSRIs). Unfortunately, these treatments are effective in just over half of diagnosed individuals, leaving a considerable population treatment-resistant. A great deal of research has therefore focused on understanding the neurobiological mechanisms that underlie the psychopathology of depression and anxiety to elucidate novel therapeutic targets. Despite important recent developments such as the use of ketamine for intractable depression, the molecular basis of the etiology and treatment of depression remains largely unknown.

Fibroblast growth factor 2 (FGF2) is a potent astroglial mitogen which is not only critical during development, but is one of few growth factors (along with brain derived nerve factor BDNF) that remain upregulated in the adult brain [3, 4]. As such, FGF2 plays a large role in the response to injury in the adult brain and neuroplastic events such as postnatal neurogenesis [5], dendritic plasticity and long-term potentiation [6–9]. More recently, FGF2 has also been implicated in anxiety and depressive behaviors, both in rodent models and in human studies [10–18]. Work from the Akil laboratory demonstrated that FGF2 shows anxiolytic properties in rats bred for high- anxiety and we have previously shown that depletion of the *FGF2* gene increases anxiety behavior and HPA activity [11–13]. Furthermore, we demonstrated that FGF2-induced changes in HPA activity were necessary for the anxiolytic effects of FGF2 [12]. In humans, FGF2 and FGF receptor levels are downregulated in post-mortem tissue of individuals that had a history of mood disorders. Furthermore, no changes in FGF2 were noted in the post-mortem brains of individuals with mood disorders that were successfully treated with anti-depressant medication prior to death [14, 15, 18, 19].

In addition to a role for FGF2 in the display of depressive and anxiety behaviors, several studies have linked FGF2 to the therapeutic effects of anti-depressant treatments such as SSRIs [20]. Central FGF2 levels increase in response to SSRI treatment in rodents [21, 22], and, in humans, mutations in the *FGF2* gene predict responsivity to SSRI treatment [23]. Importantly, these studies are mainly correlational in nature, therefore in the current study we examined whether the *FGF2* gene was necessary for the antidepressant effects of fluoxetine.

## Methods

### Experimental animals and procedure

Sixty-seven adult male FGF2 homozygous knockout (FGF2KO) and wildtype (WT) control mice (Black Swiss background; Jackson Laboratory STOCK Fgf2tm1Doe/J), were bred by establishing heterozygous breeding pairs. Wildtype control mice were comprised of 20 WT litter mates and 30 WT mice from the same background that were ordered from Charles River, Canada, and used as controls distributed across groups. Mice were single housed in standard (27cm X 17cm X 13cm), fully transparent polypropylene cages. Mice were genotyped using the primers and PCR amplification protocol as recommended by JAX (https://www2.jax.org/protocolsdb/f?p=116:5:0::NO:5:P5_MASTER_PROTOCOL_ID,P5_JRS_CODE:22270,003256). All stress mice were placed in an isolated stress room for a period of five weeks, with basic cage environment provided (nesting material and shelter). Mice were maintained on a 12-hour light/dark cycle at 21°C with ad lib food and water unless a particular stressor required otherwise (see below). Six groups were used in total; see Table 1 for further breakdown. All animal use procedures were approved by the Carleton University Committee for Animal Care, according to the guidelines set by the Canadian Council for the Use and Care of Animals in Research.

**Table 1:** Group assignment and treatment numbers.

		VEHICLE	FLUOXETINE
**CONTROL**	**Wildtype**	**Beh *n* = 10** **qPCR *n* = 10**	**Beh *n* = 10** **qPCR *n* = 10**
**STRESS**	**Wildtype**	**Beh *n* = 16** **qPCR *n* = 9**	**Beh *n* = 14** **qPCR *n* = 9**
	**FGF2KO**	**Beh *n* = 8** **qPCR *n* = 4**	**Beh *n* = 9** **qPCR *n* = 4**

#### Pilot study

13 (7 Vehicle, 6 Fluoxetine) WT and 8 FGF2KO (4 Vehicle, 4 Fluoxetine) were singly housed and maintained on a 12-hour light/dark cycle at 21°C with ad lib food and water. Behavioral results from vehicle-injected mice only were reported in a previous publication [12]. All mice received daily injections of either vehicle or fluoxetine (Prozac, Sigma-Aldrich, 10mg/kg) for three weeks prior to behavior testing.

### Chronic variable stress (CVS)

**Fig 1:**
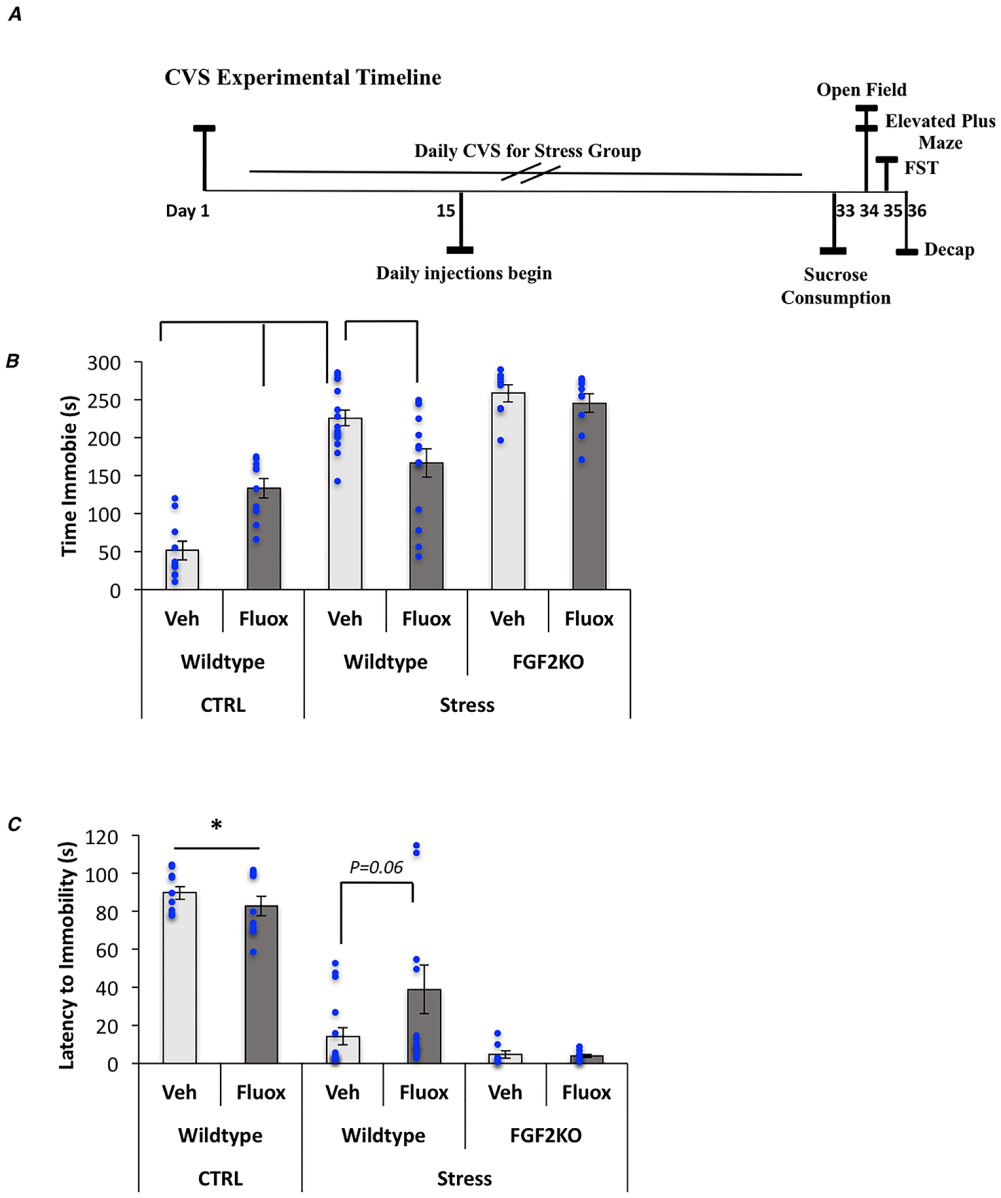
Fluoxetine reverses CVS-induced depressive behavior in WT but not FGF2KO mice. (A) Experimental timeline. (B) Graphical representation of the time spent immobile in the forced swim test. (C) Graphical representation of the latency to immobility in the forced swim test. Bars represent means ± standard error of the mean, data points reflect individual scores. Asterisks denote significant differences from all other groups.

This experiment used a CVS paradigm adapted from the Duman laboratory to induce a depressive and anxiety-like behavioral phenotype. An experimental timeline is shown in Fig 1A. All animals were exposed to various mild stressors daily over 35 days (2–3 per 24-hour period) that were uncontrollable in both time and duration to prevent habituation. Stressors consisted of the following conditions: altered light/dark cycle, food and water deprivation (overnight), isolation, cage tilt (30 degrees), exposure to odor, soiled bedding (~200 ml water added to bedding material), no bedding, swim for 10 minutes and lastly, restraint (for 15 minutes, in restraint cones that have an opening in the front for ventilation). Animals were monitored both during and immediately after each procedure. See Table 2 for an approximate breakdown of the daily stressors and duration.

**Table 2:** Chronic variable stress schedule.

DAY	STRESSORS	DURATION
1	Cage tilt, wet bedding	4hrs, overnight
2	Restraint, lights on	15min, overnight
3	Odour, Swim	5hrs, 10min
4	Cage tilt, empty cage	2hrs, overnight
5	Wet bedding, lights on	4hrs, overnight
6	Restraint, odour	15min, 4hrs
7	Cage tilt, lights on	5hrs, overnight
8	Odour, empty cage	3hrs, overnight
9	Restraint, cage tilt	15min, 3hrs
10	Swim, lights on	10min, overnight
11	Odour, wet bedding	3hrs, overnight
12	Cage tilt, empty cage	4hrs, overnight
13	Cage tilt, lights on	5hrs, overnight
14	Restraint, odour	15min, 4hrs
15	Swim, wet bedding	10min, overnight
16	Cage tilt, empty cage	3hrs, overnight
17	Restraint, odour	15min, 5hrs
18	Swim, lights on	10min, overnight
19	Wet bedding, odour	3hrs, 3hrs
20	Cage tilt, empty cage	3hrs, overnight
21	Restraint, lights on	15min, overnight
22	Cage tilt, odour	3hrs, 4hrs
23	Wet bedding, empty cage	3hrs, overnight
24	Restraint, wet bedding	15min, 4hrs
25	Odour, lights on	4hrs, overnight
26	Cage tilt, odour	3hrs, 3hrs
27	Wet bedding, restraint	4hrs, 15min
28	Odour, empty cage	5hrs, overnight
29	Cage tilt, wet bedding	4hrs, 3hrs
30	Restraint, lights on	15min, overnight
31	Cage tilt, wet bedding	3hrs, 4hrs
32	Odour, lights on	4hrs, overnight
33	Sucrose Consumption (1hr); Restraint, cage tilt	15min, 4hrs
34	Behavior Testing OF, EPM	-
35	Behavior Testing FST	-

### Antidepressant treatment and injection protocol

During the final three weeks of stress, half of the mice received the antidepressant treatment fluoxetine. All mice received intraperitoneal injections of either vehicle (sterile saline, VWR Canada) or fluoxetine (Prozac, Sigma-Aldrich, dose of 10mg/kg, dissolved in sterile saline at a concentration of 2mg/ml) daily for the final three weeks of the experiment. Mice were injected once daily at approximately the same time and monitored closely for any signs of distress or discomfort.

### Behavioral tests

All behavioral tests were conducted as per our previously-published studies [12].

#### Forced swim test

To assess the effects of CVS on learned helplessness, a depressive behavior measure, the forced swim test was used. Mice were individually placed in a glass cylinder (15.5cm diameter) that contained 10cm-deep temperature-controlled water (23–25 °C) for a period of 5 minutes. Time immobile (i.e., floating while making only necessary movements that require the animal to keep their head above the water), latency to become immobile (time at which animal first shows signs of immobility) and time spent swimming (i.e., active, horizontal movements) were recorded on a video camera and scored by an independent observer blind to conditions of the experiment. Immediately following the task, animals were dried off using a paper towel and placed back in their home cage.

#### Sucrose consumption test

The sucrose consumption test was conducted as a measure of anhedonia in the mice. Mice were habituated to a 1% sucrose solution for a 48-hour period followed by overnight water deprivation. For the test, the sucrose bottle was given back for 1-hour and bottles were weighed before and after the test. Normal ad libitum access to food was maintained throughout both habituation and testing periods. Access to water bottles resumed immediately after the test. All solutions were made fresh each day. Mice remained single housed throughout the process. The test was conducted in the final week (5) of the experiment. Results from two mice were omitted from the final analysis due to spillage of the sucrose solution prior to post-measurement.

#### Elevated plus maze

The elevated plus maze was used to measure anxiety and exploratory behaviors. The dimensions of the open arms are 30 x 5cm, with two enclosed with 25cm walls, and elevated 30cm from the floor, the room was brightly lit at 650 lux. The mouse was then recorded exploring the maze for a total of five minutes and tracked using AnyMaze Video Tracking System.

#### Open field test

A second measure of anxiety behaviour included was the open field test. Mice were placed in the corner of a brightly lit (650 lux) box (50 x 50cm x 35cm) and videotaped with AnyMaze Video Tracking System for 5 minutes. Time spent in the pre-defined zones (periphery and center) was recorded. Anxiety and exploratory behaviors were measured and included the amount of time an animal spent in the center or peripheral zone, as well as general measures of motor activity.

#### Emotionality score

The emotionality score was derived as previously described in Guilloux (2011) [24]. Z scores were calculated for each test and normalized for locomotor behavior, as appropriate.

$$\text{Z}\;\text{score}\;\text{OF}=\frac{\left(\frac{X-\mu }{\sigma }\right)TimeCenter+\left(\frac{X-\mu }{\sigma }\right)Proportion\;Peripheral\;Distance}{2}$$

$$\text{Z}\;\text{score}\;\text{EPM}=\frac{\left(\frac{X-\mu }{\sigma }\right)TimeOpen+\left(\frac{X-\mu }{\sigma }\right)Proportion\;Closed\;Distance}{2}$$

$$\text{Z}\;\text{score}\;\text{FST}=\left(\frac{X-\mu }{\sigma }\right)TIme\;Immobile$$

$$\text{Emotionality}\;\text{score}=\frac{ZscoreOF+ZscoreEPM+ZscoreFST}{3}$$

### Animal sacrifice and quantitative real time PCR

One day following completion of the experiment mice were decapitated and a subset of brains were collected, flash frozen and stored at -80°C until use. Briefly, the hippocampus was dissected and RNA was extracted using the STRATAGENE RNA isolation kit (Agilent). Quantitative real time PCR was conducted as previously described [12]. Reverse transcription was carried out using Superscript III (Invitrogen) and GR and FGF2 levels were assessed using TAQMAN assays (Life Technologies; Assay ID: Mm00433832_m1 Gene Symbol: NR3C1; Assay ID: Mm01285715_m1 Gene Symbol: Fgf2) as per the manufacturer’s instructions. All assays were conducted and analyzed using the Applied Biosystems 7500 machine and proprietary software set to detect TAQMAN probes/assays.

### Statistical analyses

All data were analyzed using IBM SPSS Statistics Data Editor (Version 20). Analysis of variance was conducted with genotype (WT vs. FGF2KO), condition (control vs. stress) and treatment (vehicle vs. fluoxetine) as independent variables. Because this model is unbalanced in its design, we applied a more conservative alpha level to reduce the likelihood of type 1 errors, thus probability values were considered significant when *p* ≤ 0.01. If the overall model was significant then ANOVAs were followed by post-hoc t-test comparisons with Bonferonni correction for non-orthogonal tests; t-tests were considered significant when *p* ≤ 0.05.

## Results

### Chronic fluoxetine treatment does not reverse the effects of CVS on depressive-like behavior in FGF2KO mice

In order to assess the effects of *FGF2* gene deletion on the ability of fluoxetine to decrease depressive-like behaviors in WT and FGF2KO mice, we conducted a pilot study comparing WT and FGF2KO mice treated with either chronic vehicle or fluoxetine. Results from both the forced swim test and the sucrose consumption test showed no effect of fluoxetine in either WT or FGF2KO mice (data not shown). Because these were experimentally naïve animals and previous studies in mice have shown that fluoxetine decreases depressive behaviors following perturbations such as stress [25], we hypothesized that exposure to chronic stress would induce depressive behaviors and therefore increase sensitivity to fluoxetine’s anti-depressant effects.

Therefore, in a subsequent study to examine the effects of *FGF2* gene deletion on fluoxetine’s ability to decrease depressive-like behaviors, WT and FGF2KO mice were exposed to CVS and tested on the forced swim and sucrose consumption tests. In addition, we had non-stressed WT mice that were similarly injected with vehicle or fluoxetine. Results from the forced swim test showed a significant overall model both for total immobility time (F_(5,66)_ = 29.26, p<0.0001) (Fig 1B) and latency to immobility (F_(5,66)_ = 28.14, p<0.0001) (Fig 1C). Comparisons of simple effects revealed that all stress groups had a significant increase in immobility from vehicle-treated, WT controls (p<0.01) (Fig 1B). In addition, WT, stressed mice that were treated with fluoxetine showed a significant decrease in immobility from their vehicle-treated WT stressed counterparts (p<0.01). FGF2KO mice showed no response to fluoxetine (p>0.05) (Fig 1B). Similarly, comparisons of simple effects revealed that all stressed groups had a significant decrease in the latency to immobility as compared to non-stressed controls (p<0.01) (Fig 1C). WT, stressed mice treated with fluoxetine showed a trend for an increase in the latency to immobility (p = 0.06). FGF2KO mice did not respond to fluoxetine treatment (p>0.05) (Fig 1C).

Results from the sucrose consumption test, a measure of anhedonia, showed no significant differences in sucrose consumption (baseline), water consumption (baseline) or sucrose/water consumption (data not shown). In addition, no differences in weight were observed across groups (data not shown) (p>0.05 for all comparisons).

### Chronic fluoxetine treatment does not reverse the effects of CVS on anxiety-like behavior in FGF2KO mice

**Fig 2:**
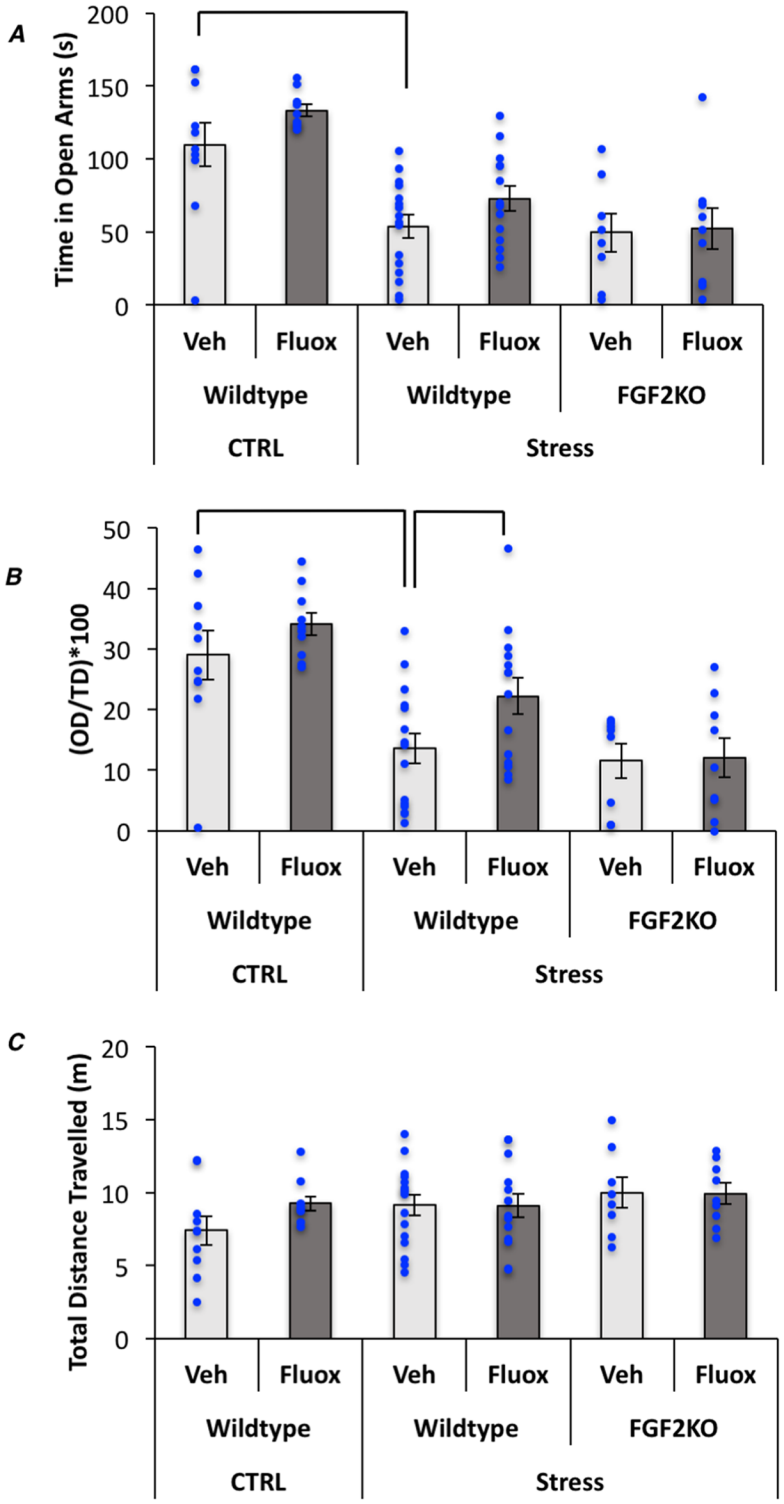
Fluoxetine reverses CVS-induced anxiety behavior in WT but not FGF2KO mice on the elevated plus maze. (A) Graphical representation of the total time spent in the open arms of the EPM. (B) Graphical representation of the distance travelled in the open arms relative to the total distance travelled in the EPM. (C) Total distance travelled over the entire EPM. Bars represent means ± standard error of the mean, data points reflect individual scores.

To examine the effects of *FGF2* gene deletion on fluoxetine’s ability to decrease anxiety-like behaviors, mice were also tested on the elevated plus maze and open field tests. Results from the elevated plus maze showed a significant overall model for time spent in the open arms (F_(5,66)_ = 10.53, p<0.0001) (Fig 2A), distance travelled in open arms (F_(5,66)_ = 5.03, p<0.001) (data not shown), and distance in open arms/total distance travelled (F = 3.8_(5,66)_, p<0.01) (Fig 2B). Simple effects showed that control mice spent more time in the open arms than all stress groups. WT stressed mice treated with fluoxetine showed an increase in the proportion of distance travelled in open arms as compared to vehicle-treated counterparts. Interestingly, no effect of fluoxetine was again observed in FGF2KOs. Finally, the overall model for total distance travelled was not significant, suggesting no changes in locomotor activity overall (F_(5,66)_ = 1.12, p>0.01) between groups (Fig 2C).

**Fig 3:**
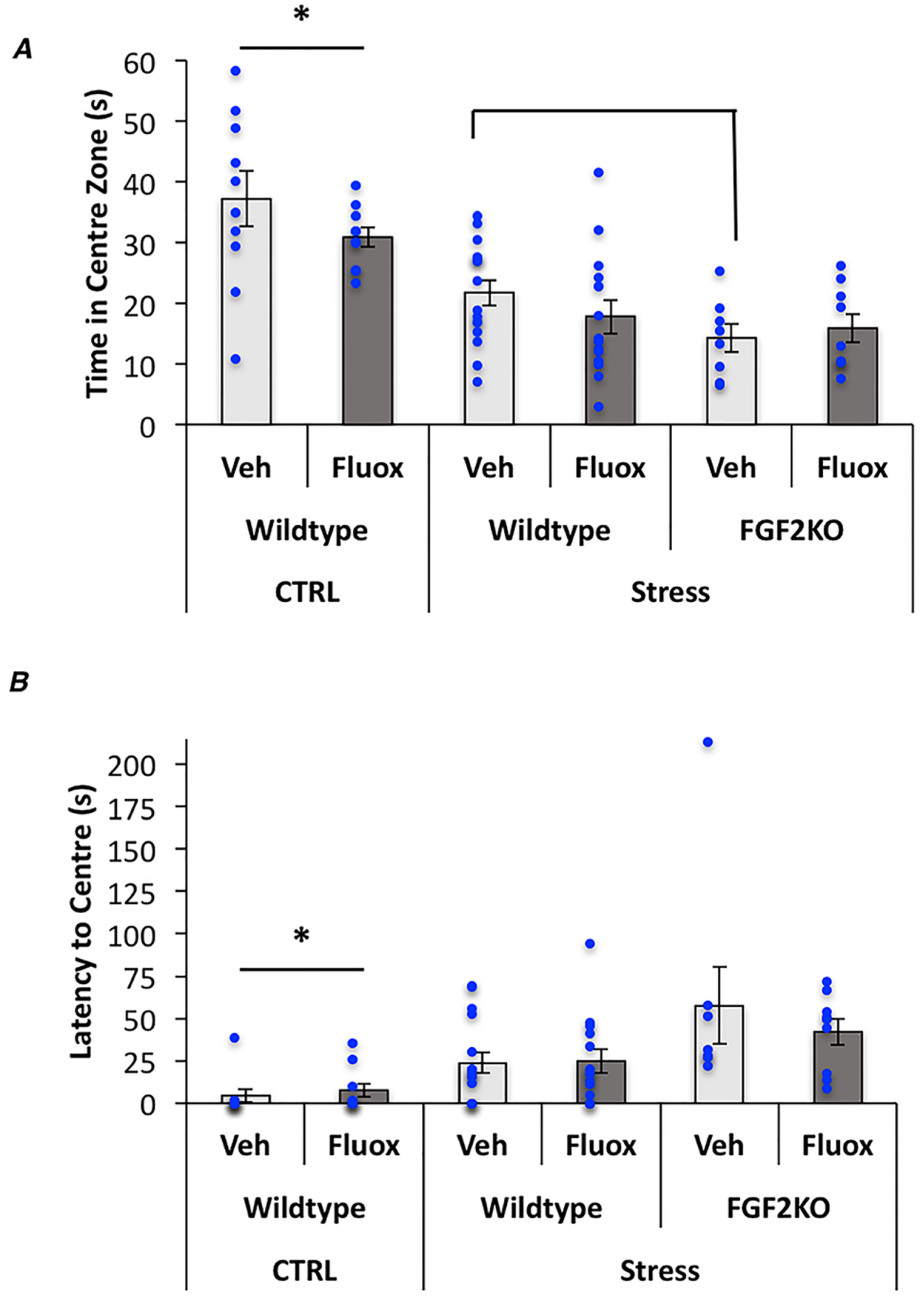
Fluoxetine has no effect on CVS-induced anxiety behavior in mice on the open field test. (A) Graphical representation of the time in the centre in the open field test. (B) Graphical representation of latency to enter the centre zone in the open field test. Bars represent means ± standard error of the mean, data points reflect individual scores. Asterisks denote significant differences from all other groups.

Results from the open field test showed a significant overall model for time in center zone (F_(5,66)_ = 9.7, p<0.0001) (Fig 3A) and latency to enter the center zone (F_(5,66)_ = 4.12, p = 0.003) (Fig 3B). Post-hoc tests showed that all stressed groups spent less time in the center and had a longer latency to enter the center. Moreover, FGF2KO mice showed a further decrease in time in the center and a trend for a longer latency (p = 0.07) as compared to their WT, stressed counterparts, suggesting an additive effect of stress and *FGF2* gene deletion. No effects of fluoxetine were observed on any measure of the open field test.

### Chronic fluoxetine treatment increases *FGF2*, but not *GR* gene expression levels in wild-type mice only

**Fig 4:**
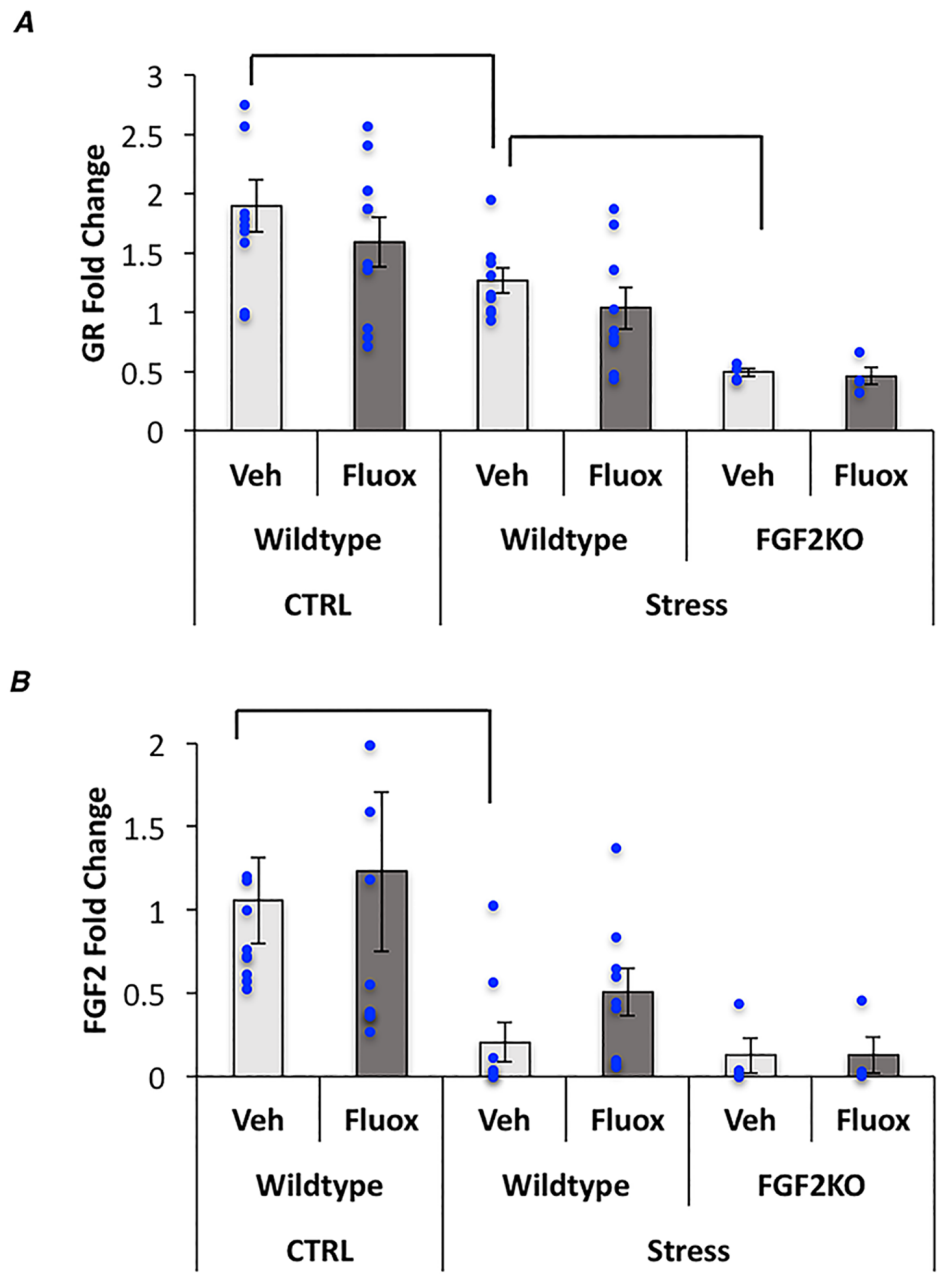
CVS decreases hippocampal GR and FGF2 expression levels. (A) Graphical representation of the GR fold change. (B) Graphical representation of the FGF2 fold change. Bars represent means ± standard error of the mean, data points reflect individual scores.

Because we have previously shown that the effects of FGF2 administration on anxiety behavior are mediated through FGF2-induced *GR* upregulation [12], we quantified hippocampal levels of *FGF2* and *GR* using q-RT-PCR. Results showed a significant overall model for *GR* as well as main effects of both stress and genotype (p<0.01 for all) such that stressed mice showed lower *GR* levels than non-stressed mice and FGF2KO mice showed a further decrease in *GR* (Fig 4A). No effects of fluoxetine were observed on *GR* (Fig 4A).

*FGF2* levels did not change overall (p = 0.04), however there were significant main effect of stress (p<0.01) such that all stressed groups had decreased levels of FGF2 from non-stressed controls (Fig 4B). Because it has been previously shown that acute administration of fluoxetine increases hippocampal FGF2 [26], we compared FGF2 levels of Vehicle treated, stressed WT mice to their fluoxetine treated counterparts and found a trend (p = 0.06) for an increase in FGF2 levels with fluoxetine treatment.

**Fig 5:**
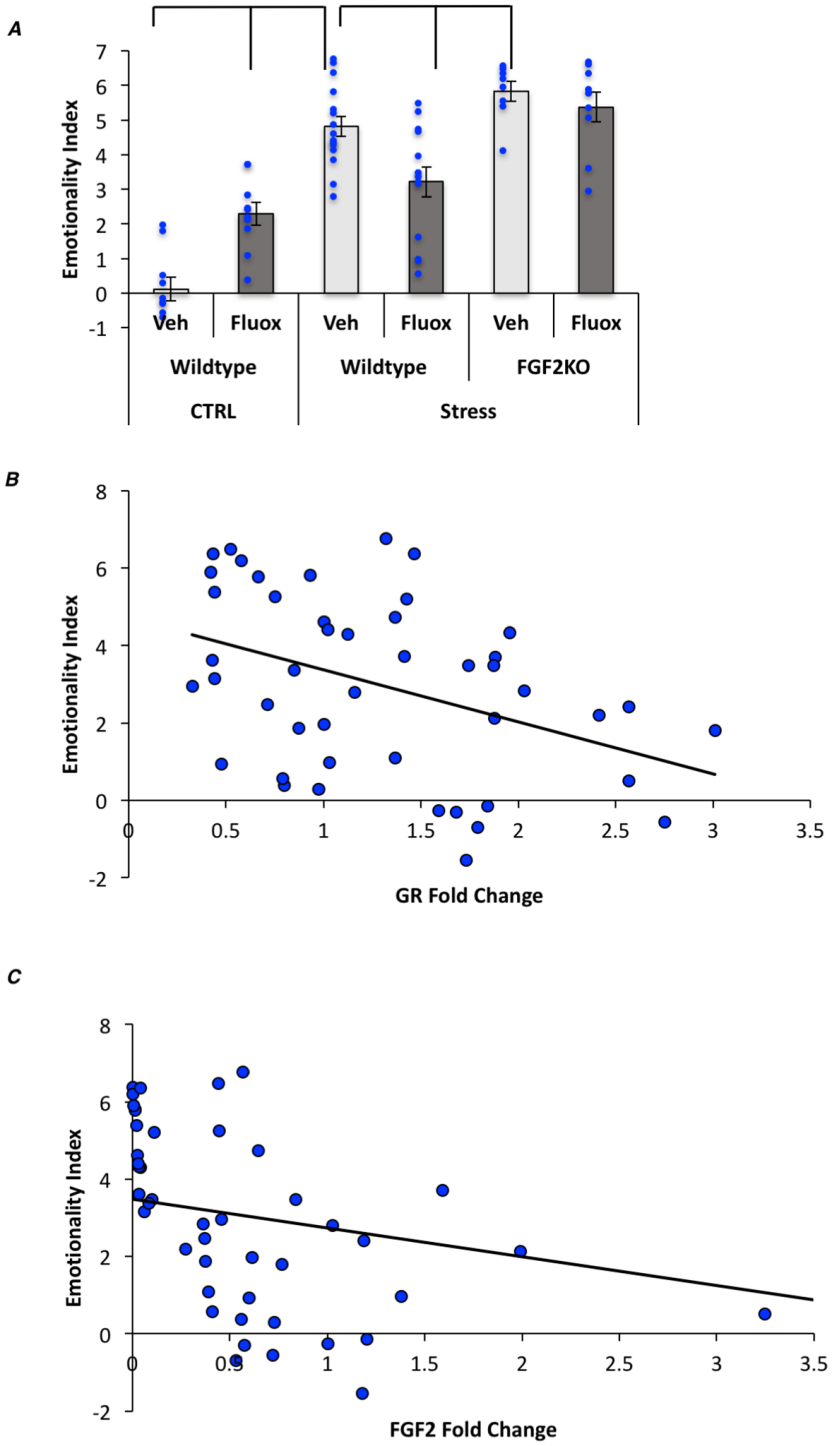
Fluoxetine reverses the CVS-induced increase in the emotionality index in WT but not FGF2KO mice. (A) Graphical representation of the emotionality index. (B) Correlation scatter plot showing the relationship between GR expression levels (fold change) and the total emotionality index. (C) Correlation scatter plot showing the relationship between the FGF2 expression levels (fold change) and the total emotionality index. Bars represent means ± standard error of the mean, data points reflect individual scores.

In order to conceptualize the overall anxiety and depressive phenotype, we included a total “emotionality score” that we have previously employed and was first described by the Sibille laboratory [24, 27]; a significant increase in emotionality was induced by stress, which was reduced in fluoxetine-treated stressed WT mice (Fig 5A). No effect of fluoxetine was seen in FGF2KOs.

Because behavior and gene expression levels were conducted in the same mice, we correlated both GR and FGF2 gene expression levels to our behavioral measures (See Table 3). Significant correlations were observed between *GR* levels and measures of the open field, elevated plus maze and forced swim test (see Table 3 for specific correlations). FGF2 levels were also significantly correlated to the elevated plus maze and forced swim tests, however no significant relationship was observed between FGF2 and the open field test (See Table 3). *GR* levels were also significantly correlated to the emotionality score (r = -0.409; p<0.01) (Fig 5B). Similarly, *FGF2* levels were correlated with the emotionality score (r = -0.302; p<0.05) (Fig 5C).

**Table 3:** Pearson correlations between GR and FGF2 fold change with depressive and anxiety-like behaviors.

	**1**	**2**	**3**	**4**	**5**	**6**	**7**
**1. Time Centre (OF)**							
**2. Centre Distance (OF)**	0.69^a^						
**3. Time Open (EPM)**	0.45 ^a^	0.46 ^a^					
**4. Open Distance (EPM)**	0.19	0.38 ^a^	0.77 ^a^				
**5. Time Immobile (FST)**	-0.54 ^a^	-0.30 ^b^	-0.43 ^a^	-0.28			
**6. Latency to Immobility (FST)**	0.55 ^a^	0.33 ^a^	0.59 ^a^	0.31 ^b^	-0.80 ^a^		
**7. GR Fold Change**	0.51 ^a^	0.36 ^b^	0.42 ^a^	0.26	-0.39 ^a^	0.41 ^a^	
**8. FGF2 Fold Change**	0.24	0.26	0.35 ^b^	0.10	-0.33 ^b^	0.37 ^b^	0.36^b^

^a^ Where *p* < 0.001 (2-tailed)

^b^ Where *p* < 0.05 (2-tailed)

## Discussion

The current study sought to ascertain whether there is a causal role for the FGF2 gene in mediating the therapeutic effects of the SSRI fluoxetine in mice exposed to CVS. No effects of fluoxetine were observed in naïve mice, however, fluoxetine predictably reversed many of the stress-induced depressive and anxiety behaviors in WT mice. Remarkably, FGF2KO mice did not show any reversal of stress-induced behaviors with fluoxetine, suggesting that *FGF2* is necessary for the therapeutic effects of fluoxetine. These findings corroborate studies in humans with major depressive disorder that showed that variants of the FGF2 gene can predict efficacy of SSRI treatment and suggest an important role for FGF2 in the mechanisms of anti-depressant action [23]. While the anti-depressant and anxiolytic effects of FGF2 have been previously documented in several rodent models, the current study would suggest that FGF2 not only has antidepressant/anxiolytic properties, but that it is necessary for fluoxetine’s therapeutic effects.

Interestingly, somewhat discrepant results were observed on measures of anxiety behavior, as expected, fluoxetine decreased anxiety in WT mice on the elevated plus maze, however fluoxetine had no significant effect on anxiety in WT mice in the open field test. Although both the open field and elevated plus maze are used as measures of anxiety, discrepancies between the findings of these two tests have been previously reported [28]. Moreover, previous reports have shown strain differences that are consistent with our results here in C57/Bl6 mice [29]. The reason for the dissociation between these two tests remains unknown, nevertheless, *FGF2* gene deletion did not show any responses to fluoxetine on either of these tests, suggesting that regardless of the behavioral output measured, *FGF2* is necessary for the effects of fluoxetine.

Fluoxetine decreased the total emotionality score in CVS-exposed mice as expected. However, a paradoxical effect of fluoxetine was also noted in non-stressed, wild-type control mice, where fluoxetine actually increased the emotionality score. It is not clear if this is a genuine increase in emotionality; given the limited number of studies that have included this index, it is not possible to examine if this is consistent. However, because locomotor activity is a component in generating the emotionality score, it is possible that fluoxetine-induced locomotor activity changes contributed to the change in emotionality index in this group. Further studies will be needed to determine if this is a consistent effect or a limitation in this index.

We have previously shown that the effects of FGF2 administration on anxiety behavior were mediated through FGF2’s upregulation of hippocampal glucocorticoid receptor expression [12]. In the current study, glucocorticoid receptor expression was similarly decreased in FGF2KO mice, however, expression did not change with fluoxetine administration in WT mice, suggesting that changes in expression of hippocampal glucocorticoid receptors cannot be the FGF2-dependant mechanism of action by which fluoxetine reverses stress-induced behavioral changes, at least at the time-point examined. Importantly, however, this does not preclude the possibility that the reduction in GR caused by *FGF2* gene deletion is responsible for the lack of response to fluoxetine in FGF2KO mice. Interestingly, it appears as if GR levels show an additive response to stress and *FGF2* gene deletion and indeed, GR levels were significantly correlated with all behavioral tests. Although we have previously shown that GR is causally implicated in the anxiolytic effects of FGF2 [12], whether GR is causally related to the effects of chronic stress in the current study remains to be determined.

Although FGF2 has been widely implicated in anxiety and depressive-like behavior, FGF2 levels in response to the CVS model currently employed have not been previously measured. Interestingly, hippocampal FGF2 levels decrease in WT mice following chronic stress to similar levels observed in FGF2KO mice, and levels of FGF2 were negatively correlated with most of the behavioral measures tested, further strengthening the relationship between hippocampal FGF2 and anxiety and depressive-like behavior.

There are many known effects of FGF2 in the adult brain which are related to neuroplastic events. Like FGF2, there are several other growth factors that are not downregulated post-development and continue to be expressed in the adult brain, such as BDNF and VEGF. Importantly, these factors also play critical roles in regulating neuroplasticity and have been implicated in anxiety and depressive-like behaviors and their treatment [30]. While it is not clear how each of these growth factors uniquely contributes to modulating depressive and anxiety-like behaviors, unlike BDNF and VEGF, FGF2 is largely produced by astroglial cells, and the effects of FGF2 on neuroplasticity are typically mediated through astroglial cells [31]. This suggests that FGF2’s unique contribution to the pathology and treatment of anxiety and depressive-like behaviors may be better understood by examining the cells that are targeted, astroglia. Interestingly, a large body of evidence has begun to implicate astroglia in depressive/anxiety phenotypes [24, 32–38], including work demonstrating that an injection of a gliatoxin and not a neurotoxin into the prefrontal cortex, induces a phenotype comparable to that observed following CVS [39]. It is therefore possible that the FGF2-mediated effects of fluoxetine that we have observed here are related to FGF2-induced astroglial changes rather than changes in HPA activity. Indeed, chronic fluoxetine treatment has been demonstrated to increase the number of hippocampal astroglial cells as marked by S100B and GFAP, increase the production of anti-inflammatory cytokines and reverse stress-induced decreases in astroglial specific proteins associated with gap-junction connectivity [33, 40].

Whether astroglia, astroglial functions, or HPA changes are the downstream target of FGF2 mediated changes induced by fluoxetine remains to be determined, however, the current study reaffirms the potential of FGF2 as a novel therapeutic target in the treatment of depression and anxiety disorders.

## Supporting information

S1 File PLOS one raw data excel file. (XLSX)
